# Authenticating the Geographical Origin of Jingbai Pear in Northern China by Multiple Stable Isotope and Elemental Analysis

**DOI:** 10.3390/foods13213417

**Published:** 2024-10-26

**Authors:** An Li, Duoyong Zhao, Jiali Li, Jianping Qian, Qiusheng Chen, Xun Qian, Xusheng Yang, Jie Zhao

**Affiliations:** 1 Institute of Quality Standard and Testing Technology, Beijing Academy of Agriculture and Forestry Sciences, Beijing 100097, China; lionlian@126.com (A.L.);; 2 Institute of Quality Standards & Testing Technology for Agro-Products, Xinjiang Academy of Agricultural Sciences, Urumqi 830091, China; 3 Institute of Agricultural Resources and Regional Planning, Chinese Academy of Agricultural Sciences, Beijing 100081, China; 4 Institute of Agro-Product Safety and Nutrition, Tianjin Academy of Agricultural Sciences, Tianjin 300381, China; tjzbscqs@126.com; 5 Institute of Biotechnology and Food Science, Hebei Academy of Agriculture and Forestry Sciences, Shijiazhuang 050031, China

**Keywords:** stable isotope, multielement, pear, geographical origin, chemometrics

## Abstract

The Jingbai pear is one of the best pear species in China with high quality and nutrition values which are closely linked to its geographical origin. With the purpose of discriminating the PGI Mentougou Jingbai pear from three other producing regions, the stable isotope ratios and elemental profiles of the pears (*n* = 52) and the corresponding soils and groundwater were determined using isotope ratio mass spectrometry (IRMS) and inductively coupled plasma mass spectrometry (ICP-MS), respectively. The results revealed that δ^15^N, δ^18^O_J_, and Li were significantly different (*p* < 0.05) in samples from different regions, which indicated their potential to be used in the geographical origin classification of the Jingbai pear. The nitrogen isotopic values of the pear pulp were positively correlated with the δ^15^N value and nitrogen content of the corresponding soils, whilst the B, Na, K, Cr, and Cd contents of the pear pulps were positively correlated with their corresponding soils. Orthogonal partial least squares discriminant analysis (OPLS-DA) was performed in combination with analysis of the stable isotopes and elemental profiles, making it possible to distinguish the cultivation regions from each other with a high prediction accuracy (a correct classification rate of 92.3%). The results of this study highlight the potential of stable isotope ratios and elemental profiles to trace the geographical origin of pears at a small spatial scale.

## 1. Introduction

Ascertaining the geographical origin of food is crucial to enhancing its quality and security, particularly for high-value agrofoods with a protected designation of origin label [1]. A total of 2495 food products in China have been approved as geographical indication (GI) products nationwide [2]. One of the highest-quality GI products is the Jingbai pear, a variety of *Pyrus ussuriensis* Maxima pears that is traditionally grown in northern China. The Jingbai pear shows relatively high levels of soluble solids compared with most of other pear cultivars grown in China [3]. The Mentougou district of Beijing, where the Jingbai pear has been farmed for over 400 years, is home to the protected region of GI products [4]. There has been growing concern among consumers of high-quality foods regarding their origin, including those with GI certification. However, misleading food origin labelling occurs in the market due to the motivation of economic gains. Appropriate analytical techniques are needed in this regard to safeguard honest producers and prevent consumers from being duped.

Various indicators, such as stable isotopes [5,6,7], multielements [8,9], organic components [10,11], ^1^H-nuclear magnetic resonance (^1^H-NMR) [12,13], and nontargeted metabolite profiling [14,15] have been used in conjunction with chemometrics to determine the geographical origin of foods. Stable isotope (δ^13^C, δ^15^N, δ^2^H, and δ^18^O) and multielemental analysis via IRMS and ICP-MS, respectively, were thought to be the most promising methods amongst the many approaches to confirming the origin of an object [16]. Carbon isotopic composition generally reflects on the photosynthetic pathway of plants, including C_3_, C_4_, and crassulacean acid metabolism (CAM), according to their ability to uptake atmospheric CO_2_ [17]. Climate and soil variables also play important roles in carbon isotope discrimination due to their effects on photosynthesis according to a global study [18]. Nitrogen isotopes can be used to determine agricultural practices, and more specifically, the source of plant nutrients. Organic fertilisers that have undergone denitrification and lost their lighter ^14^N isotope are higher in ^15^N than synthetic fertilisers made via the Haber process, which normally have low quantities of ^15^N [19]. The isotope composition of precipitation water was found to exhibit an altitude and a continental effect [20]. Specifically, the heavier ^2^H and ^18^O isotopes gradually become more depleted as elevation increases or whilst travelling inland from coastal regions [21]. Consequently, during fruit development and maturity, water with varying δ^2^H and δ^18^O values (uptake from precipitation and groundwater) from various locations is transferred into the fruit [22]. With the exception of certain halophytes, plant roots typically do not fractionate isotopes when absorbing water [23]. However, given transpiration, water in plant tissues contains larger quantities of ^2^H and ^18^O than the source water [24]. Furthermore, it is likely that the result of biochemical fractionation will be that substantial isotope fractionation happens during the transfer of hydrogen and oxygen atoms from water to the creation of organic compounds [25]. However, whether the hydrogen and oxygen isotopes in organic compounds or water contribute to distinctions between different geographical origins remains unclear. A growing number of studies have examined many elements as markers to ascertain the origin of fruit geographically [26,27,28]. The distribution of elements in soil differs between locations, and this variation may persist in the cultivated plant, which accounts for the successful application of multiple elements in the geographical origin of food [29].

This study focuses on the use of stable isotope ratio and multielement analysis to verify the geographical origin of the Jingbai pear in a small-scale production region in northern China. We aim to describe the relationship between various factors (e.g., geography, climate, and soil) and indicators (stable isotopes and multielements) and their potential and limitations in terms of establishing the geographical origin of the Jingbai pear in a small-scale farming region. The overall objective is to propose a reliable implementation strategy for stable isotope ratio and multielement analysis in the determination of the geographical origin of the Jingbai pear.

## 2. Materials and Methods

### 2.1. Sampling

**Figure 1:**
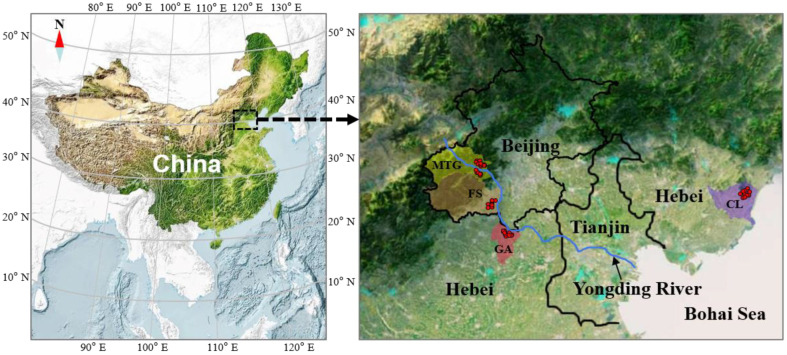
Map of Jingbai pear collection sites from four regions in northern China.

A total of 52 samples of Jingbai pear were procured from 45 primary plantation regions situated across four regions in northern China (MTG, Mentougou District, Beijing; FS, Fangshan District, Beijing; GA, Gu’an County, Hebei Province; CL, Changli County, Hebei Province) during the harvest period of 2021 (Figure 1). Ordinarily, we collected one sample of more than 3 kg of pear fruit from one plantation. For some large plantations, two samples were collected. Groundwater and soil from 0 to 40 cm were also collected in these regions. The geo-climatic parameters in the four regions are presented in Table 1.

### 2.2. Sample Preparation

#### 2.2.1. Pear

The pear sample was crushed into pulp. Approximately 2 g of pulp were transferred into a glass bottle with absorbent cotton in the headspace for water extraction on a BJJL-2200 fully automatic vacuum condensation extraction system (Beijing Jianling Technology Co., Ltd., Beijing, China). The rest of the pulp was freeze-dried in a vacuum freeze dryer, homogenised by grinding and sieving in 80 meshes, and the solid powder samples were stored in a desiccator.

#### 2.2.2. Soil

The soil was naturally dried and then ground and sieved through a 40-mesh sieve. Groundwater was filtered through a 0.45 μm microporous filter membrane before IRMS analysis.

### 2.3. Stable Isotope Ratio Analysis

#### 2.3.1. Carbon and Nitrogen Isotope Ratio Measurement

Approximately 0.25 and 5.0 mg of the dried powdered pear were weighed into tin capsules (8.0 mm × 5.0 mm) for the determination of δ^13^C and δ^15^N, respectively. For the analysis of δ^15^N in the soil, approximately 30 mg of the soil was weighed into tin capsules (8.0 mm × 5.0 mm). δ^13^C and δ^15^N were determined using an isotope ratio mass spectrometer (ThermoFisher Scientific MAT 253, Bremen, Germany) equipped with an elemental analyser (Flash HT2000). The samples were combusted at 960 °C in a combustion reactor. A helium carrier gas with a purity of 99.999% was set to 100 mL min^−1^ [30]. The isotopic data were calibrated with USGS40 (L-glutamic acid, U.S. Geological Survey, Reston, VA, USA; δ^13^C = −26.39‰, δ^15^N = −4.52‰) and USGS41a (L-glutamic acid, U.S. Geological Survey, Reston, VA, USA; δ^13^C = 36.55‰, δ^15^N = 47.55‰). The standard deviations (*n* = 5) for the determination of δ^13^C and δ^15^N using the above method were no more than 0.10‰ and 0.15‰, respectively.

#### 2.3.2. Hydrogen and Oxygen Isotope Ratio Measurement

The groundwater and extracted juice water were filtered through a 0.45 μm microporous membrane and then transferred into a glass vial before EA-IRMS measurement [22]. Besides the juice water, the hydrogen and oxygen isotopes of the dried pulp were also measured to investigate the geographical traceability of the isotopes from different sources. About 0.2 mg of the dried powdered pulp samples was placed into a silver capsule (5.0 mm × 3.5 mm) for hydrogen and oxygen isotope analysis. To eliminate the interference of the exchangeable hydrogen, the organic reference materials (USGS54, USGS55, and USGS56) made of wood and the pear samples in unsealed silver capsules were placed together in a desiccator for 5 days and then left open in the laboratory for 3 days to balance exchangeable hydrogen in the organic matrix with ambient water vapour [30]. The H and O isotopes of the samples were analysed in a reactor tube at 1380 °C and a column oven at 75 °C. The helium carrier gas (99.999% purity) was set to 100 mL min^−1^. The isotopic data of the solid samples were calibrated with USGS54 (Canadian lodgepole pine, U.S. Geological Survey, Reston, VA, USA; δ^2^H = −150.4‰; δ^18^O = 17.79‰), USGS55 (Mexican ziricote, U.S. Geological Survey, Reston, VA, USA; δ^2^H = −28.2‰; δ^18^O = 19.12‰), and USGS56 (South African red ivory wood, U.S. Geological Survey, Reston, VA, USA; δ^2^H = −44.0‰; δ^18^O = 27.23‰), whilst the isotopic data for water were calibrated with VSMOW (Vienna Standard Mean Ocean Water, IAEA, Vienna, Austria; δ^2^H = 0‰; δ^18^O = 0‰), USGS45 (Biscayne aquifer drinking water, U.S. Geological Survey, Reston, VA, USA; δ^2^H = −10.3‰; δ^18^O = −2.238‰), and USGS47 (Lake Louise Water, U.S. Geological Survey, Reston, VA, USA; δ^2^H = −150.2‰; δ^18^O = −19.80‰). The standard deviations (*n* = 5) for the determination of δ^2^H_water_, δ^18^O_water_, δ^2^H_solid_, and δ^18^O_solid_ using the above method were no more than 2.0‰, 0.50‰, 3.0‰, and 0.30‰, respectively.

#### 2.3.3. IRMS Data

The isotope ratios were expressed as relative to the international standards mentioned above and denoted in delta notation in accordance with the following formula [31]:

(1) $$\delta =\frac{R_{\mathrm{sample}}-R_{\mathrm{reference}}}{R_{\mathrm{reference}}}\times 1000‰$$

where R_sample_ indicates the ^13^C/^12^C, ^15^N/^14^N, ^2^H/^1^H, and ^18^O/^16^O of an unknown sample, and R_reference_ denotes the ^13^C/^12^C, ^15^N/^14^N, ^2^H/^1^H, and ^18^O/^16^O of the international reference standards, respectively. The δ^13^C values were expressed as relative to Vienna Pee Dee Belemnite (V-PDB), δ^15^N to air, whilst δ^2^H and δ^18^O were expressed relative to SMOW.

### 2.4. Sample Digestion and ICP-MS Measurement

Powdered pear (0.5 g) and soil (0.1 g) were treated with a microwave-assisted digestion process utilising a MARS 6 classic microwave digester (CEM Co., Matthews, NC, USA) operating at a constant power of 1600 W. The sample was placed in a Teflon reactor with 5 mL of 95% HNO_3_ and kept for 1 h. The reactor was then put in a microwave oven set to temperature control for 30 min. The digested sample was cooled and subsequently diluted to 50 mL with deionised water before ICP-MS (Agilent 7500 Series, Agilent Technologies, Inc., Santa Clara, CA, USA) analysis [22]. For multipoint calibration and method validation, mixed standard solutions (Agilent 8500-6940, Agilent 8500-6942 and Agilent 8500-6948, Agilent Technologies, Inc., USA) including the 22 analytical elements (Na, Mg, K, Ca, Li, B, Al, Ti, V, Cr, Mn, Fe, Co, Ni, Cu, Zn, As, Se, Cd, Sb, Ba, and Pb) were diluted into various concentrations. The spiking experiment revealed that all 22 element calibration curves had acceptable linearity (R^2^ > 0.995) in their ranges and the element recoveries in the pear and soil fell between 84.9% and 119.7%, respectively. This outcome demonstrated the validity of the entire analysis procedure for elemental analysis.

### 2.5. Statistical Analysis

A one-way analysis of variance (ANOVA) followed by a Bonferroni test was used to study the differences in the samples that are related to their geographical origin, which were considered statistically significant at a *p*-value ≤ 0.05 by using the general linear model of SPSS (version 18.0). The correlations between the variables were analysed via a Pearson correlation test by the software Origin (version 2021). OPLS-DA was applied via Soft Independent Modelling of Class Analogy (SIMCA) (version 13.0) in the classification of the pear samples in accordance with their geographical origin.

## 3. Results and Discussions

### 3.1. Characteristics of Multiple Stable Isotopes

**Figure 2:**
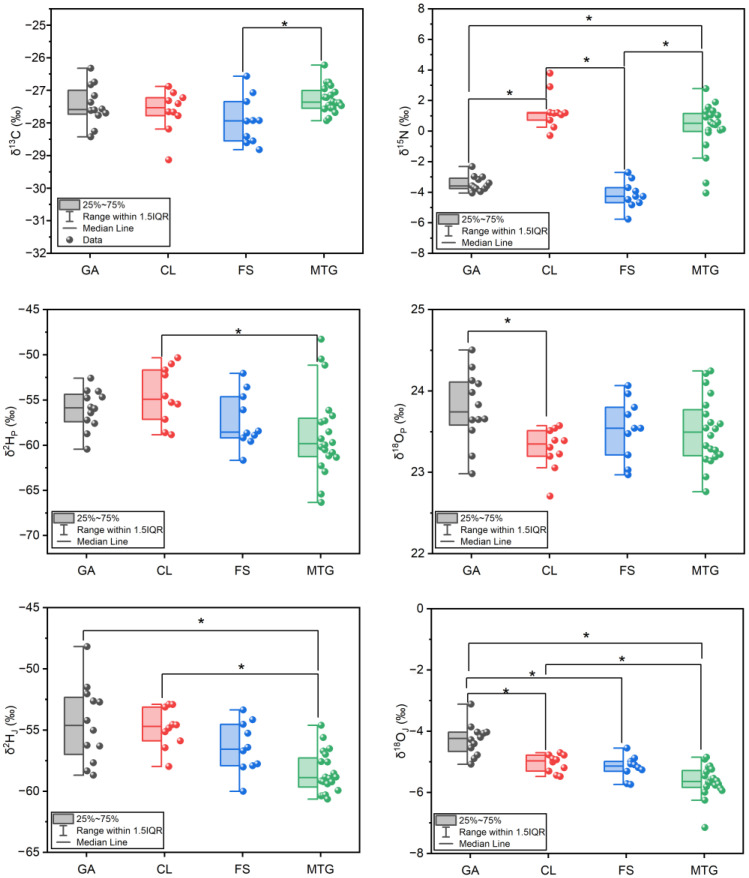
Box plots of multiple stable isotopic values in pears from four producing areas. The symbol ‘*’ indicates significant differences at *p* < 0.05 between different regions using the Bonferroni test.

**Figure 3:**
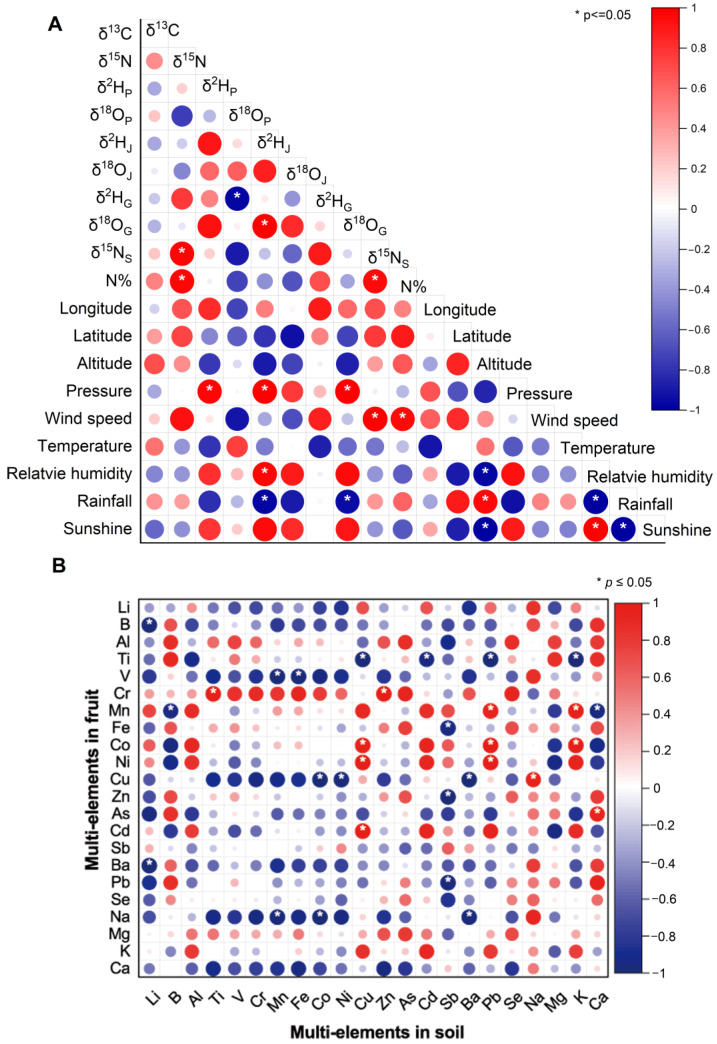
Correlation analysis between geographical indicators and various other factors. (**A**) The stable isotope and geoclimatic factors; (**B**) the multielements in pears and their cultivated soil. The symbol ‘*’ indicates a significant correlation; the blue colour denotes a negative correlation, and the red colour denotes a positive correlation; the size of dot indicates numerical size of the absolute correlation coefficient.

Analytical results of multiple stable isotopes in Jingbai pears collected from four different regions are illustrated in Figure 2. Moreover, the correlation between the isotopic ratios in the fruit with various geographical climate factors are displayed in Figure 3A. The δ^13^C values of Jingbai pears from different regions were between −29.13‰ and −26.23‰, indicating a C_3_ pathway of photosynthesis during the carbon fixation of the pear tree [32,33]. The only significant difference in the δ^13^C value amongst the four regions was observed between the MTG and FS samples, with mean values of −27.3% and −27.9%, respectively. The narrow distribution of the δ^13^C values of Jingbai pears of different origins is not surprising given the small spatial scale of the cultivated regions. In addition, the photosynthetic pathway and environmental factors including temperature, pressure, and sunshine all play roles in the carbon isotope fractionation in plant tissues. However, no significant correlations were found between the various factors and the carbon isotope (Figure 3A). This is probably due to the fact that all of these regions have similar climates, meaning that no single factor is primarily responsible for the small variations in the carbon isotopic compositions of these samples.

The δ^15^N values of all the pear samples ranged from −5.8 to 3.8‰. Similar ranges of variation were previously reported in other fruits such as mango (from −3.65 to 7.91‰) [34] and avocado (from −2.4 to 6.4‰) [35]. As shown in Figure 2, significant differences were found for the δ^15^N values amongst these four regions. Generally, the samples from MTG and CL showed higher δ^15^N values in comparison with the samples from GA and FS. Nutrients in the soil are the only source of nitrogen for most plants except for rhizobial nitrogen-fixing plants [36]. Therefore, the δ^15^N value of the pear samples depends on the nitrogen isotope of the soil background and the type of fertiliser used for the cultivation of the plant [37]. As shown in Figure 3A, significant correlations (*p* < 0.05) were observed between the δ^15^N values of the pears and the factors of the δ^15^N values of the soil and the nitrogen quantity in the soil. Variations in the δ^15^N values of the pears and the corresponding soil for the four regions are most likely due to the differences in fertiliser type. The geological conditions might also be one of the reasons that lead to the significant differences in the δ^15^N values of these samples. As shown in Figure 1, compared with the plain areas of FS and CL, the sampling points in MTG and CL are closer to mountainous areas. Previous studies have reported that soil organic matter (SOM) increases with elevation because the average temperature drops with altitude, slowing the decomposition of SOM and leading to the accumulation of SOM and soil nitrogen at higher elevations [38,39].

Hydrogen and oxygen isotopic compositions in agrofood are recognised as good geographical indicators due to the strong relationships between the isotopic values and water and the local environment [40]. In this research, we studied the hydrogen and oxygen stable isotopes of water from the fruit juice (δ^2^H_J_ and δ^18^O_J_) and the organic matter from the pulp (δ^2^H_P_ and δ^18^O_P_). The isotopic values of oxygen for the juice water and pulp ranged from −7.15 to −3.11‰ and from 22.71 to 24.50‰, respectively. Significant enrichment of ^18^O was observed from the pear juice water to the pear pulp, as shown in Figure 2. This finding is consistent with the previous literature on other fruits and vegetables, where δ^18^O values in juice ranged from −6.9 to 10.4‰ and 16.2 to 36.6‰, respectively, as reported by Wu et al. [41]. This result suggests that the transpiration mechanism enriches leaf water with ^18^O, making the water entering photosynthesis isotopically different from the water taken up by the plant [42]. However, the hydrogen isotopic composition of the pear pulp was not ^2^H-enriched as supposed. Compared with the δ^2^H values of the groundwater (from −73.48 to −49.41‰), the isotopic values of hydrogen varied between −66.34 and −48.28‰ in the juice water of the pears. And, in the pulp, they varied between −60.64‰ and −48.19‰. Similar findings also reported that the isotopic values of hydrogen from peach juice water collected from northern China varied between −53.43 and −29.58‰, showing they were more enriched than the groundwater (from −67.00 to −54.60‰) [22]. However, the small variation in hydrogen isotopic values between juice water and pulp is probably due to the complicated fractionation of ^2^H during the Calvin cycle reaction. For instance, the transferable hydrogen in nicotinamide adenine dinucleotide hydrogen (NADH) in the peroxisome is enriched with ^2^H due to transhydrogenation, forming a nicotinamide adenine dinucleotide phosphate hydrogen (NADPH) interconversion between NADH and NADPH [43], whilst isomerization between dihydroxyacetone phosphate (DHAP) and glyceraldehyde-3-phosphate (GAP) causes strong ^2^H-enrichment because the carbon–bond hydrogen can exchange H with the surrounding water [44]. The δ^2^H_P_ and δ^2^H_J_ values of the MTG samples were significantly lower than the samples from CL, indicating a continental effect of hydrogen isotope in precipitation, which is the initial source of hydrogen in fruit. The δ^2^H_J_ and δ^18^O_J_ values from GA were higher than those in the samples from other regions. This result is reasonable because GA is located at a lower latitude than the other regions. The δ^18^O_P_ values observed in the samples did not show any continental or latitude effect.

### 3.2. Multielemental Compositions

Table 2 presented the findings of 22 mineral element analyses conducted on Jingbai pear samples collected from four cultivation regions. The outcomes of ANOVA analysis indicate a significant (*p* < 0.05) variability in the multielemental composition of pear samples amongst the four areas. Notably, the levels of elements ranking from K, Mg, Ca, and Na were found to be very high in pear samples. The observed result is quite similar to a previous study on Chinese ‘Cuiguan’ pears reported by Zeng et al. [45]. The cumulative total of these four macro elements accounts for 99.04% of the overall element content detected in this research. The samples from CL exhibit the highest average content of Li elements. By contrast, the samples from MTG exhibit the lowest average content of Li elements. A heat map (Figure 3B) that displays the multielemental contents of Jingbai pears and the soil they were grown in may more thoroughly show relationships between variables and migration mechanisms from soil to pear. Specific elements in the pears, such as B, Na, K, Cr, and Cd, have a positive but not statistically significant correlation with the soil, according to our correlation analysis. According to Reimann et al. [46], the elemental composition of a plant is generally not simply reflected by the underlying soil, and the plant/soil system appears highly nonlinear due to the complicated element input system of plants. The small spatial scale might also be one of the reasons that led to the nonsignificant correlations of most element concentrations between soil and plant [47].

### 3.3. Discrimination of Geographical Origin of the Jingbai Pear by Chemometric Models

**Figure 4:**
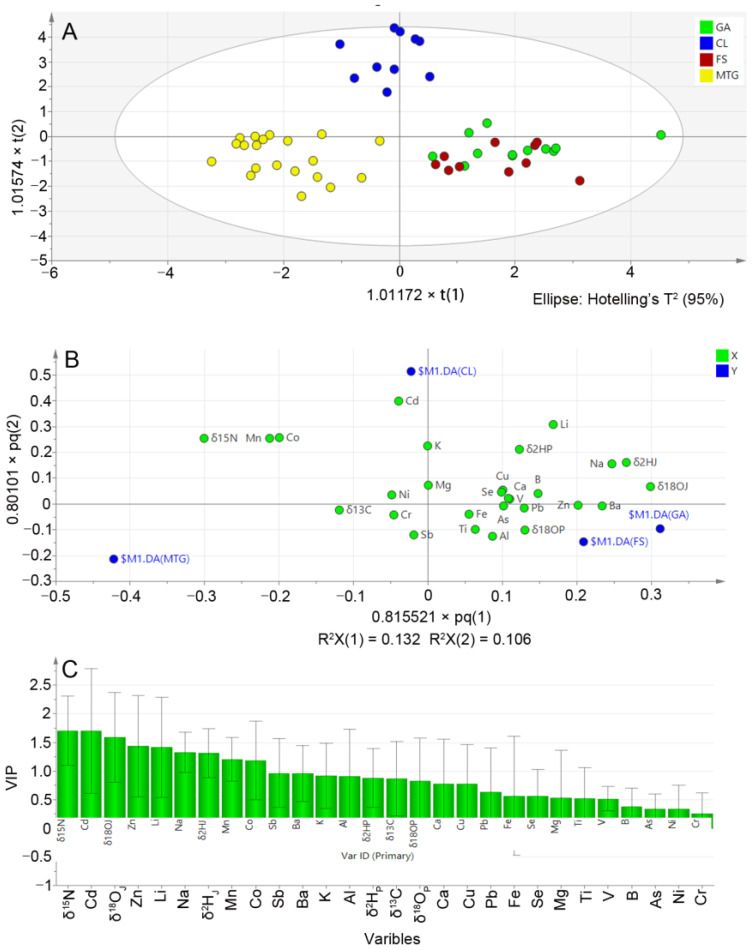
OPLS-DA modelling of the stable isotope and multielement signatures of pears collected from different regions in China: (**A**) Score plots into the PredC1–PredC2 planes and (**B**) loading plots of 28 variables into the PredC1–PredC2 planes. (**C**) The VIP plots of variables.

OPLS-DA works well for classifying data with noisy and multicollinear variables, which are typical of many biological data types [48]. It is also used to clarify which variable contains the information that contributes to the classification [49]. Here, we established an OPLS-DA model through SIMCA, and a total of three predictive components (PredC) were obtained. The predictive capacity (Q^2^) for the three components were 0.28, 0.24 and 0.12, respectively, with the Q^2^(cum) of 0.64, indicating an adequate level of predictability for this model. The classification model by OPLS-DA allowed a clear differentiation of each group of pear samples in a 2D plot on the basis of the first two principal components (Figure 4A). According to the score and loading charts (Figure 4A,B), the loading distribution of variables shows that δ^15^N, Co, and Mn were more representative of the differentiation between the MTG samples and the others. Cd contributed most to the distinction between the CL samples and the others, whilst δ^2^H_J_ and δ^18^O_J_ contributed most to the separation between samples from GA, FS, and the others.

The variable importance of projection (VIP) plot showed that the most important discriminant variables (VIP value > 1) were δ^15^N, Cd, δ^18^O_J_, Zn, Li, Na, δ^2^H_J_, Mn, and Co (Figure 4C). The cross-validation discriminant accuracies of Jingbai pears from four regions were 91.7%, 100%, 70.0%, and 100% (Table 3), suggesting a relatively high classification probability. Jingbai pears grown in GA could be classified as originating from FS (8.3%, 1/12), whilst Jingbai pears from FS were misclassified as coming from the GA region (30.0%, 3/10). The Jingbai pears from CL and MTG were 100% classified. The average of the right classification was 92.3% for the OPLS-DA model by employing the stable isotope and multielement signatures of the Jingbai pears. Therefore, the proposed method is reliable and applicable for the identification of the geographical origin of Jingbai pear samples.

## 4. Conclusions

In this study, stable isotopes and multielemental compositions of Jingbai pears from four different regions were measured and used to establish a statistical model for geographical authentication. An OPLS-DA model using four stable isotopes and 22 elements was used to confirm the origin of Jingbai pears from the different regions. δ^15^N, Cd, δ^2^H_J_, δ^18^O_J_, Zn, Li, Co, Mn, Na, K, and Al had the best discrimination ability to differentiate Jingbai pears from different regions. The discriminant accuracy for PGI Jingbai pears from MTG with those from other regions was 100%. This study found that the average discriminant accuracy for Jingbai pears was 92.3%, which is useful for regulatory verification purposes considering the complexities of agricultural production across the regions and the potential risk of deliberate mislabelling of Jingbai pears from different regions to improve economic gains. Overall, stable isotopes and multielements combined with chemometric analysis provide an effective method to determine the geographical origins of Jingbai pears and verify the authenticity of PGI-labelled Jingbai pears.

## Figures and Tables

**Table 1:** Region and climate data of the producing area of the collected Jingbai pear samples.

Region	Mentougou (MTG) (*n* = 20)	Fang Shan (FS) (*n* = 10)	Gu’an (GA) (*n* = 12)	Changli (CL) (*n* = 10)
Longitude (°)	116°0′25″–116°7′44″	116°10′41″–116°11′55″	116°21′50″–116°22′23″	119°11′25″–119°13′46″
Latitude (°)	39°57′28″–40°0′36″	39°36′6″–39°38′28″	39°25′31″–39°25′59″	39°45′30″–39°45′48″
Altitude (m)	150–472	34–38	26–29	18–71
Pressure ^1^ (hpa)	996.3	1000	1003.5	1004.4
Wind speed ^2^ (m/s)	1.8	1.5	1.3	1.9
Temperature ^3^ (°C)	25.4	25.2	25.4	24.9
Humidity ^4^ (%)	76.8	80.6	82.4	81.3
Sunshine hours ^5^ (h)	292.2	328.6	337.6	331.3
Precipitation ^6^ (mm)	782.4	545.4	410.4	487.6

^1^ average atmosphere pressure of the two months before cultivation; ^2^ average wind speed of the two months before cultivation; ^3^ average daily temperature of the two months before cultivation; ^4^ average daily relative humidity of the two months before cultivation; ^5^ cumulative sunshine hours of the two months before cultivation; ^6^ cumulative precipitation of the two months before cultivation.

**Table 2:** Multicomparison ANOVA of multielemental contents of Jingbai pear samples collected from different regions (mg/kg).

Elements	Regions			
	GA (*n* = 12)	CL (*n* = 10)	FS (*n* = 10)	MTG (*n* = 20)
Na	54.50 ± 8.71 b ^1^	50.86 ± 9.08 b	42.04 ± 11.93 ab	29.55 ± 16.50 a
Mg	571.50 ± 208.16 a	645.69 ± 66.50 a	670.69 ± 193.67 a	611.39 ± 112.48 a
K	6663.49 ± 599.82 a	7577.51 ± 438.21 b	7128.25 ± 1102.51 ab	6829.04 ± 645.07 ab
Ca	294.62 ± 99.38 a	246.71 ± 54.76 a	229.26 ± 72.20 a	228.02 ± 62.22 a
Li	0.12 ± 0.06 ab	0.22 ± 0.13 c	0.13 ± 0.08 b	0.04 ± 0.03 a
B	16.0 ± 7.2 a	14.7 ± 2.0 a	15.9 ± 6.2 a	13.8 ± 3.1 a
Al	7.2 ± 2.1 ab	6.40 ± 1.49 a	12.7 ± 10.6 b	7.95 ± 2.19 ab
Ti	1.05 ± 0.30 a	0.79 ± 0.26 a	1.05 ± 0.43 a	0.95 ± 0.39 a
V	0.28 ± 0.09 a	0.24 ± 0.12 a	0.23 ± 0.17 a	0.18 ± 0.17 a
Cr	2.80 ± 2.57 a	3.07 ± 2.40 a	3.40 ± 2.19 a	3.35 ± 1.58 a
Mn	6.61 ± 0.60 a	11.21 ± 3.00 b	6.12 ± 0.98 a	9.45 ± 3.37 b
Fe	27.94 ± 8.45 a	29.55 ± 8.84 a	35.63 ± 22.07 a	27.16 ± 8.54 a
Co	0.08 ± 0.03 a	0.14 ± 0.03 b	0.08 ± 0.03 a	0.11 ± 0.03 ab
Ni	1.23 ± 0.99 a	1.62 ± 0.93 a	1.18 ± 0.91 a	1.31 ± 0.84 a
Cu	4.04 ± 0.69 a	4.06 ± 1.34 a	3.61 ± 1.13 a	3.05 ± 1.25 a
Zn	2.05 ± 0.33 a	2.15 ± 0.98 a	3.49 ± 1.33 b	1.62 ± 0.47 a
As	0.13 ± 0.06 a	0.11 ± 0.06 a	0.14 ± 0.10 a	0.10 ± 0.11 a
Se	0.07 ± 0.06 a	0.08 ± 0.05 a	0.10 ± 0.09 a	0.06 ± 0.06 a
Cd	0.003 ± 0.001 a	0.03 ± 0.02 b	0.001 ± 0.001 a	0.004 ± 0.002 a
Sb	0.03 ± 0.03 a	0.01 ± 0.01 a	0.01 ± 0.01 a	0.03 ± 0.03 a
Ba	3.75 ± 1.03 b	2.83 ± 1.34 ab	3.55 ± 1.78 b	2.09 ± 1.19 a
Pb	0.14 ± 0.06 a	0.12 ± 0.06 a	0.18 ± 0.17 a	0.10 ± 0.06 a

^1^ Symbols bearing different letters (a–c) in the same row are significantly different (*p* < 0.05).

**Table 3:** Classification of Jingbai pear samples via OPLS-DA.

Region	N	GA	CL	FS	MTG	Correct
GA	12	11	0	1	0	91.7%
CL	10	0	10	0	0	100%
FS	10	3	0	7	0	70.0%
MTG	20	0	0	0	20	100%
Total	52	14	10	8	20	92.3%

## Data Availability

The original contributions presented in the study are included in the article, further inquiries can be directed to the corresponding author.
